# Evaluation of the Saudi Healthy Eating Index

**DOI:** 10.3389/fnut.2026.1814949

**Published:** 2026-07-10

**Authors:** Haya M. Alzeer, Fatima Adel Almadani, Malikah Aldawsari, Ghadeer S. Aljuraiban, Reem Alsukait

**Affiliations:** Department of Community Health Sciences, College of Applied Medical Sciences, King Saud University, Riyadh, Saudi Arabia

**Keywords:** diet quality, Diet Quality Index, dietary assessment, Healthy Eating Index, Saudi Arabia

## Abstract

**Background:**

The Saudi Healthy Eating Index (SHEI) was developed to evaluate adherence to the Saudi Healthy Plate Guide 2024. This study aimed to conduct a preliminary assessment of the psychometric properties of the SHEI.

**Methods:**

A secondary analysis was conducted using data from a cross-sectional study on 702 Saudi adults in Riyadh and Al-Madinah regions. Dietary data were collected using multiple-pass 24-h recalls, along with anthropometric and sociodemographic information. The SHEI was evaluated for content validity, concurrent validity, construct validity, internal consistency reliability, and component correlations.

**Results:**

The SHEI showed strong concurrent validity with the HEI-2015 (*r* = 0.867, *p* < 0.001). The SHEI also demonstrated evidence of construct validity, with total scores averaging 52.64 ± 11.05 and clear variation across individuals and score quintiles (*p* < 0.001). Smokers had significantly lower scores (48.8 ± 0.1) compared to non-smokers (53.3 ± 0.5), while differences by sex, education level, and family income were modest and non-significant. The index correlated weakly with energy intake (*r* = −0.103, *p* = 0.006) and yielded moderate to high scores for exemplary menus. It also revealed five underlying dietary dimensions, accounting for 57.39% of total variance. However, internal consistency was low (Cronbach’s *α* = 0.237), and correlations among components ranged from moderate to weak.

**Conclusion:**

The findings support the validity of the SHEI but indicate limited reliability. Future validity and reliability studies of the SHEI should aim for better representation by addressing sample size and food database limitations.

## 1 Introduction

Diet quality, measured through Diet Quality Indices (DQIs), is closely linked to health outcomes (1). Low diet quality has been associated with higher risks of overweight/obesity, diabetes, cardiovascular disease, cancer, and mortality (2–4). Beyond individual health, DQIs support public health monitoring by identifying variations in dietary patterns and highlighting nutritionally vulnerable groups (5–10). These indices can also inform food security and nutrition policy efforts (11).

Despite their extensive international application, studies using DQIs to assess diet quality within Saudi Arabia are limited. Existing research assessing adherence to different Saudi dietary guidelines has used simplified food-frequency questionnaire-based tools reflecting the Healthy Food Palm (2013) or the Saudi Healthy Plate Guide (SHPG) 2020, but these tools were developed for individual studies, used varying scoring criteria, and were applied only to specific population subgroups. The limited validation and reliability testing of these tools further represent a significant limitation (12–16). In the absence of a Saudi-specific validated and standardized tool, several studies have instead used indices based on other dietary guidelines (14, 17, 18). Developing a Saudi-specific DQI is essential to ensure that diet quality is assessed in alignment with national guidelines and local dietary habits in a standardized manner. Therefore, the Saudi Healthy Eating Index (SHEI) was developed to measure adherence to the SHPG-2024 (19). The SHEI consists of 12 components that produce a total score of 0–100, reflecting the SHPG-2024 dietary recommendations across fruits, vegetables, grains and whole grains ratio, protein foods and plant-based proteins, dairy, beverages, fatty acid, saturated fat, added sugar, and sodium.

Psychometric evaluation is an essential step in the development of DQIs (1), as it ensures that such tools yield accurate, consistent, and meaningful results (20). Physical measurements, such as weight or temperature, have objective reference points, making reliability and validity evaluation straightforward, in contrast to measurement tools for intangible concepts such as diet quality or quality of life (20). While the specific methodological approaches may vary between DQIs, the core evaluation components consistently include construct validity, content validity, concurrent validity, and internal consistency reliability (21–25). The aim of this study was to conduct a preliminary assessment of the psychometric properties of the Saudi Healthy Eating Index (SHEI) through comprehensive validation and reliability testing, to be used as a diet quality assessment tool for Saudi adults.

## 2 Methods

### 2.1 Data sources

#### 2.1.1 Participant-level data collection

This analysis utilized data from a cross-sectional study conducted between 2021 and 2023 among Saudi adults in the Riyadh and Al-Madinah regions (26). Ethical approval was obtained from the King Saud University Institutional Review Board (No. 21/0319/IRB). Adults from the general population were recruited through social media platforms, and those who expressed interest were scheduled for an in-person visit. Study details were explained to all participants, informed consent was obtained, and participants were informed of their right to withdraw.

Saudi adults aged ≥18 years who were generally healthy were eligible to participate. A total of 1,074 individuals completed the study procedures. Those who used nutritional supplements, antihypertensive, corticosteroid, or antidepressant medications, hormone therapy, or had a previous history of cardiovascular disease, stroke, type 1 diabetes, or dyslipidemia, as well as pregnant or lactating women and non-Saudi nationals, were excluded. Following these exclusions, participants with incomplete or incompatible dietary data were also removed, resulting in a sample of 702 individuals.

Dietary data were collected by trained clinical dietitians using two multiple-pass 24-h recalls, one in person during recruitment and another by mobile phone on a non-consecutive day. During the in-person visit, the same dietitians obtained anthropometric measurements and conducted structured interviews to collect sociodemographic and lifestyle information.

#### 2.1.2 Exemplary menus

Three sample menus were graded using the SHEI, two from national sources: the SHPG-2024 by the Saudi National Nutrition Committee, and the Calorie Guide for Weight Loss by the Saudi Ministry of Health (27–29). In addition to an international source, the DASH diet-based menu from the US National Heart, Lung, and Blood Institute, which was previously used in the HEI-2015 evaluation (21, 28).

### 2.2 Dietary data processing

All foods and beverages consumed were entered, and nutrient intakes were analyzed using ESHA’s Food Processor® Nutrition Analysis software, which provided energy and nutrient data. However, to generate SHEI scores based on food group components, a customized Excel-based dietary entry system was developed. This system incorporated the Food Patterns Equivalents Database (NHANES 2017–March 2020 Pre-pandemic version) for standardized food coding (30), and the What’s in the Foods You Eat Search Tool (2021–2023) for portion-size conversion, both derived from the USDA FoodData Central database (31). The integration of these two complementary databases enabled automated conversion of household measures (e.g., cups, spoons) to gram weights through dropdown lists linked to FPED food codes and their corresponding 37 USDA Food Patterns components. Intake was averaged across the two 24-h recalls or based on a single recall when only a single day was collected. After dietary data entry, the automated SHEI calculator embedded in the spreadsheet generated the final component and total scores for each participant. The full SHEI component definitions, cut-points, and scoring rules are reported in the index development paper (19). The HEI-2015 was scored within the same Excel-based system as the SHEI, using the HEI-2015 scoring standards (21).

Since no Saudi-specific equivalent to the FPED exists, all reported food items were categorized according to their level of correspondence with FPED entries to verify its adequacy for coding Saudi dietary data: (1) traditional foods with a close FPED match, (2) traditional foods with no close match (broken down into nutritionally similar ingredients), and (3) exact or non-traditional foods (e.g., common Middle Eastern or international items).

### 2.3 Psychometric evaluation plan

The psychometric evaluation plan (Table 1) follows established evaluation protocols for similar dietary indices, with specific methods to assess content, construct, and concurrent validity, as well as internal consistency and component correlations.

**Table 1:** Psychometric evaluation plan for the validity and reliability of the SHEI.

Evaluation item	Analysis question	Analysis strategy
Validity		
Content validity	Does the SHEI reflect the various key aspects of diet quality specified in the SHPG-2024?	Map the SHEI components to SHPG-2024 recommendations to confirm coverage (8)
Construct validity	Does the SHEI vary sufficiently among individuals?	Examine the distribution of total SHEI scores across the study population
	Does a higher SHEI score link with a lower exposure to confirmed dietary risk factors among the target population?	Compare SHEI component scores and food group intakes across quintiles of total SHEI scores
	Does the index distinguish between groups with known differences in diet quality?	Compare SHEI scores of males and females, smokers and non-smokers, people with higher and lower education levels, and higher and lower income levels using between-group comparisons
	Does the index assess diet quality independent of diet quantity?	Estimate the correlations between total and component scores with energy intake
	Is the index multidimensional, and how many dimensions does it have?	Use principal component analysis to estimate the underlying structure of the index
	Does the index give high scores to recommended menus published by trusted sources?	Compute SHEI scores for exemplary menus
Concurrent validity	Is the SHEI consistent with other validated dietary indices?	Analyze correlations between SHEI and HEI-2015 scores for the same population
Reliability		
Internal consistency	Is the index internally consistent?	Calculate Cronbach’s α
Component correlations	Are the SHEI components appropriately correlated?	Calculate correlations between each of the component scores
	Which component has the biggest impact on the total score?	Calculate correlations between each component and the sum of all others

### 2.4 Statistical analysis

All statistical analyses were performed using SPSS version 29.0.1.0 (IBM Corp., Armonk, NY). Total SHEI scores demonstrated normal distribution and were therefore reported as mean ± standard deviation (SD), and as mean ± standard error (SE) when comparing mean total SHEI scores across quintiles. However, SHEI components and continuous sociodemographic variables showed non-normal distributions, so they are reported as median (Interquartile Range (IQR)). Categorical variables were reported as n (%). Statistical significance was set at *p* < 0.05.

To evaluate construct validity, participants were divided into quintiles based on total SHEI scores. Because individual component scores were not normally distributed, trends across quintiles were assessed using the Jonckheere–Terpstra test, with results presented as median (IQR) and p for trend. Between-group comparisons were conducted using independent t-tests and one-way ANOVA for total SHEI scores. Spearman’s rank correlation coefficients were calculated to examine the relationships between energy intake (non-normally distributed) and both total SHEI scores and individual component scores. Principal component analysis was conducted after checking the required assumptions, including linearity, sample size adequacy, and continuous variables (32). Statistical tests confirmed the data were suitable for principal component analysis [The Kaiser–Meyer–Olkin (KMO) measure was 0.573, indicating marginal suitability for exploratory analysis, while Bartlett’s test was significant (*p* < 0.001)]. Components that explained meaningful amounts of variation (eigenvalues >1) were retained, and relationships were considered strong when loadings were >0.5 in the rotated component matrix. To assess concurrent validity, Pearson’s correlation coefficient was calculated between SHEI and HEI-2015 scores.

For reliability assessment, internal consistency was assessed using Cronbach’s alpha (*α*). Component correlations were evaluated using Spearman’s correlation to examine relationships between total SHEI scores, component scores, and residual scores.

## 3 Results

### 3.1 Characteristics of the sample

The sample consisted of 702 participants, of whom 62% were female, with a median age of 27 (21–38) years. The median Body Mass Index (BMI) was 25.79 (22–30.41) kg/m^2^, and the waist-to-hip ratio was 0.79 (0.72–0.89). Regarding education and income, the majority of participants held a higher education degree (i.e., Bachelor’s degree or above) (71.1%), and most reported a monthly family income above 20 k SAR (35.3%). Smoking was reported by 15.8% of participants, with a notably higher proportion among males (30.3%) compared to females (7.1%). The details are presented in Table 2.

**Table 2:** Characteristics of the study sample.

Characteristic	Median (IQR)		
	Total (*n* = 702)	Male (*n* = 264)	Female (*n* = 438)
Age (years)	27 (21–38)	25 (21–38)	27 (22–37.25)
Weight (kg)	69.4 (57.78–83.6)	80.15 (67.3–93.52)	64.40 (54.78–76.83)
Height (cm)	163 (158–171)	173 (169–178)	159.70 (156–163)
BMI (kg/m^2^)	25.79 (22–30.41)	26.93 (22.4–30.8)	25.4 (21.56–29.9)
Waist-to-hip ratio	0.79 (0.72–0.89)	0.89 (0.83–0.95)	0.74 (0.7–0.8)

	*N* (%)		
Education			
Basic education	22 (3.1%)	2 (0.3%)	20 (2.8%)
Secondary education	181 (25.8%)	79 (11.3%)	102 (14.5%)
Higher education	499 (71.1%)	183 (26.1%)	316 (45%)
Monthly family income			
Less than 10 k SAR	217 (30.9%)	74 (10.5%)	143 (20.4%)
Between 10 k-20 k SAR	237 (33.8%)	98 (14%)	139 (19.8%)
More than 20 k SAR	248 (35.3%)	92 (13.1%)	156 (22.2%)
Smokers	111 (15.8%)	80 (30.3%)	31 (7.1%)

Focusing on dietary entries, the percentage of participants with two 24-h recalls was 573 (81.6%). Among all food items from the dietary entries, 9.81% were categorized as Saudi traditional foods: 3.47% lacked close matches in the FPED, while 6.34% had nutritionally similar matches.

### 3.2 Content validity

Content validity was addressed during index development by mapping the set of SHEI components to the key SHPG-2024 recommendations to confirm coverage, as reported previously (19).

### 3.3 Construct validity

#### 3.3.1 Variability among the sample

The mean SHEI total score was 52.64 ± 11.05 (SD). As presented in Table 3, the SHEI scores showed variability across the study population, with mean (±SE) total scores ranging from 37.4 ± 0.4 in the lowest quintile to 68.2 ± 0.5 in the highest quintile (*p* < 0.001). Most components showed significant trends across quintiles, indicating that higher total SHEI scores were associated with better compliance with SHPG-2024 dietary recommendations. Specifically, the following components showed the greatest improvements across quintiles: fruits, vegetables, and saturated fat. Several components showed limited variation. Some components maintained near maximum scores across all quintiles, such as grains, protein foods, and beverages. On the other hand, sodium scores remained low across all quintiles, indicating low adherence to SHPG-2024 sodium recommendations. All components showed significant trends of at least (*p* < 0.005), except for grains (*p* = 0.904).

**Table 3:** SHEI components across total SHEI score quintiles.

Component	Quintiles of scores					
	Q1 (*n* = 141)	Q2 (*n* = 140)	Q3 (*n* = 140)	Q4 (*n* = 140)	Q5 (*n* = 141)	*P* for trend
Total SHEI score/100*	37.4 ± 0.4	46.5 ± 0.2	52.6 ± 0.1	58.5 ± 0.2	68.2 ± 0.5	
Fruit/10	0 (0–2.3)	0.8 (0–4.6)	2.8 (0–8.7)	5.5 (1.2–10)	10 (6.5–10)	<0.001
Vegetables/10	2.8 (0.4–5.2)	4.1 (2.1–6.7)	6.4 (3–10)	7 (3.5–10)	10 (5.1–10)	<0.001
Grains/5	4.8 (3.5–5)	5 (3.5–5)	5 (3.8–5)	5 (3.8–5)	4.7 (3.3–5)	0.904
Whole grains ratio/5	0 (0–0.6)	0 (0–1.2)	0.2 (0–1.2)	0.7 (0–2.4)	1.2 (0–3.3)	<0.001
Protein foods/5	5 (3.4–5)	5 (4.2–5)	5 (4.9–5)	5 (4.8–5)	5 (5–5)	<0.001
Seafood, beans, and legumes/5	0 (0–0)	0 (0–1.7)	0 (0–2.7)	0 (0–5)	3.2 (0–5)	<0.001
Dairy/10	4.4 (0.9–9.1)	6.5 (3.5–10)	4.8 (1.7–10)	6 (2.6–10)	7.1 (3.7–10)	0.004
Beverages/10	8.3 (6.7–9.8)	9.1 (7.7–10)	9.5 (8.6–10)	10 (8.7–10)	10 (8.8–10)	<0.001
Fatty acid ratio/10	0 (0–0)	0 (0–1.5)	0 (0–3.6)	0.7 (0–4.3)	5 (0–9.5)	<0.001
Saturated fat/10	1.6 (0–5.3)	3.5 (0–6.1)	5.2 (2.8–7.8)	6.5 (4–8.5)	7.6 (5.6–10)	<0.001
Added sugar/10	5.7 (2.7–8.6)	7.6 (5.6–9.9)	8.7 (6.6–10)	9.8 (8–10)	10 (8.4–10)	<0.001
Sodium/10	0 (0–2.8)	0.3 (0–3.3)	0 (0–2.8)	0.1 (0–4.1)	1.9 (0–5.4)	<0.001

*Total SHEI score reported as mean ± SE.

Component scores are reported as median (IQR) due to non-normal distribution.

#### 3.3.2 Expected differences in SHEI scores between subgroups

There is a statistically significant difference in SHEI scores between smokers and non-smokers (*p* < 0.001). The mean difference is 4.55 points, with non-smokers demonstrating higher scores (53.3 ± 0.5) than smokers (48.8 ± 0.1). Other factors showed differences in scores, but did not reach significance. Females showed a slightly higher mean score (52.8 ± 0.5) compared to males (52.3 ± 0.7), though this difference was not statistically significant (*p* = 0.509). Education level showed a gradual increase in scores from basic education (50.4 ± 2.3) through secondary education (51.6 ± 0.9) to higher education (53.1 ± 0.3), though these differences were not statistically significant (*p* = 0.175). Similarly, family income groups increased gradually from less than 10 k (51.5 ± 0.8) to between 10 k-20 k (52.4 ± 0.7), and highest in the more than 20 k group (53.8 ± 0.7), but these differences did not reach statistical significance (*p* = 0.067). Table 4 presents SHEI total and component scores across different sample subgroups.

**Table 4:** SHEI total and component scores across demographic and socioeconomic characteristics.

Component	Sex		Smoking status		Education level			Family income group		
	Males	Females	Non-smokers	Smokers	Basic education	Secondary education	Higher education	Less than 10 k SAR	Between 10 k-20 k SAR	More than 20 k SAR
SHEI Score/100*	52.3 ± 0.7	52.8 ± 0.5	53.3 ± 0.5	48.8 ± 0.1	50.4 ± 2.3	51.6 ± 0.9	53.1 ± 0.5	51.5 ± 0.8	52.4 ± 0.7	53.8 ± 0.7
Fruit/10	2.8 (0–9.6)	3.6 (0–9.3)	3.8 (0–9.5)	1.4 (0–7.5)	3.7 (0–7.5)	3.3 (0–9.9)	3.1 (0–9.3)	3.5 (0–9)	3.1 (0–9.5)	3.6 (0–9.7)
Vegetables/10	4.4 (1.8–7.9)	6.1 (3.1–10)	5.7 (2.8–10)	4.3 (1.7–8.8)	4.9 (2.3–9.2)	5 (2.3–9.7)	5.7 (2.5–10)	4.9 (2.3–10)	5.6 (2.4–9.9)	6 (2.7–10)
Grains/5	5 (3.6–5)	4.9 (3.5–5)	5 (3.6–5)	4.9 (3.4–5)	4.4 (2–5)	5 (3.5–5)	5 (3.6–5)	5 (3.5–5)	4.9 (3.5–5)	5 (3.8–5)
Whole grains ratio/5	0 (0–1.4)	0.3 (0–1.7)	0.4 (0–1.7)	0 (0–1.1)	0.5 (0–1.3)	0 (0–1.3)	0.4 (0–1.7)	0.1 (0–1.3)	0.3 (0–1.6)	0.4 (0–1.7)
Protein foods/5	5 (5–5)	5 (4–5)	5 (4.5–5)	5 (5–5)	4.3 (2–5)	5 (4.3–5)	5 (4.7–5)	5 (4.2–5)	5 (4.7–5)	5 (4.7–5)
Seafood, beans, and legumes/5	0 (0–4)	0 (0–3.2)	0 (0–3.6)	0 (0–2.6)	0 (0–1.8)	0 (0–3.1)	0 (0–3.6)	0 (0–3.7)	0 (0–2.8)	0 (0–3.7)
Dairy/10	5.7 (2–10)	6.1 (2.4–10)	6.1 (2.6–10)	4.5 (1.3–9.6)	7.9 (3.1–10)	5 (1.7–10)	6 (2.3–10)	5.9 (2.7–10)	5.9 (1.9–9.9)	6.1 (2.3–10)
Beverages/10	9.2 (8.1–10)	9.4 (8.2–10)	9.4 (8.3–10)	8.7 (7.4–10)	10 (9.3–10)	9.1 (7.8–10)	9.3 (8.3–10)	9.2 (8–10)	9.6 (8.3–10)	9.3 (8.3–10)
Fatty acid ratio/10	0.1 (0–5)	0 (0–4)	0 (0–4.2)	0 (0–5.8)	0 (0–0.9)	0 (0–5)	0 (0–4.3)	0 (0–4.3)	0 (0–4.3)	0.4 (0–4.7)
Saturated fat/10	5.8 (2.6–8.9)	4.9 (1.1–7.2)	5.1 (1.4–7.8)	5.7 (2–7.9)	4.9 (1.1–7.4)	5.5 (1.7–8.1)	5.2 (1.4–7.8)	5.3 (1.5–7.8)	5.3 (0.8–7.8)	5.3 (2.3–7.8)
Added sugar/10	9.2 (6.6–10)	8.4 (6–10)	8.7 (6.3–10)	7.7 (4.8–10)	9.3 (5.7–10)	8.4 (6–10)	8.7 (6.2–10)	8.1 (5.2–10)	9.3 (6.6–10)	8.7 (6.4–10)
Sodium/10	0 (0–2.7)	0.7 (0–4.3)	0.2 (0–4.1)	0 (0–2.8)	0.8 (0–4.7)	0.8 (0–3.5)	0 (0–3.9)	0.8 (0–4)	0 (0–3.8)	0 (0–3.7)

*Reported as mean ± SE.

Component scores are reported as median (IQR).

#### 3.3.3 Association with energy intake

To assess whether the SHEI measured diet quality independently of diet quantity, the correlation between SHEI scores and energy intake was examined (Table 5). Due to the non-normal distribution of energy intake, Spearman’s correlation was used. Results showed a weak, negative correlation between SHEI total scores and energy intake (*r* = −0.103, *p* = 0.006). This weak correlation with energy intake is consistent with the SHEI measuring diet quality rather than diet quantity. As for SHEI components, vegetables had the strongest correlation with energy intake, but this relationship was weak (*r* = −0.160, *p* < 0.001).

**Table 5:** Spearman correlation coefficients between total and component SHEI scores and energy intake with 95% confidence intervals (CI).

Component	Correlation coefficient with energy intake	*p*-value	95%CI
Total SHEI score	−0.103	0.006	[−0.175, −0.027]
Fruit	−0.069	0.068	[−0.144, 0.011]
Vegetables	−0.160	<0.001	[−0.238, −0.082]
Grains	−0.092	0.015	[−0.168, −0.017]
Whole grains ratio	−0.018	0.626	[−0.093, 0.053]
Protein foods	−0.048	0.202	[−0.126, 0.031]
Seafood, beans, and legumes	0.085	0.024	[0.012, 0.157]
Dairy	0.008	0.823	[−0.074, 0.083]
Beverages	−0.071	0.061	[−0.145, 0.004]
Fatty acid ratio	−0.057	0.129	[−0.131, 0.013]
Saturated fat	−0.041	0.280	[−0.112, 0.035]
Added sugar	0.017	0.658	[−0.057, 0.091]
Sodium	0.089	0.018	[0.016, 0.168]

#### 3.3.4 Multidimensionality

Five components had eigenvalues above 1, together explaining 57.39% of the total variation in the SHEI. As the KMO measure was marginal (0.573), this five-dimensional structure and the explained variance should be interpreted as tentative. The rotated component matrix (Table 6) shows the loading patterns of SHEI components. Component 1 explained 13.68% of the variance and was characterized by high loadings from saturated fat (0.775), dairy (−0.733), and fatty acid ratio (0.648). Component 2 explained 12.95% of the variance and was dominated by added sugar (0.763), beverages (0.730), and sodium (−0.538). Component 3 explained 10.57% of the variance and was characterized by protein foods (0.787) and seafood, beans, and legumes (0.772). Component 4 explained 10.16% of the variance and was characterized by whole grains ratio (0.660), fruit (0.584), and sodium (0.569). Component 5 explained 10.03% of the variance and was characterized by grains (−0.732) and vegetables (0.726). Overall, the five components reflected distinct dietary patterns: Component 1 suggested a general fat-related pattern, Component 2 captured an added-sugars and beverages pattern, Component 3 represented protein-rich foods, Component 4 reflected a whole-grains and fruits pattern, and Component 5 indicated variation between grain-based foods and vegetables. Sodium loaded above 0.5 on two components (0.569 on Component 4 and −0.538 on Component 2), which complicates the interpretation of these two patterns.

**Table 6:** Component loadings for the SHEI dimensions.

Component	Dimension				
	1 (13.68%)	2 (12.95%)	3 (10.57%)	4 (10.16%)	5 (10.03%)
Fruit	0.181	0.153	0.016	**0.584**	0.080
Vegetables	0.002	0.218	−0.009	−0.076	**0.726**
Grains	−0.109	0.122	−0.019	−0.049	**−0.732**
Whole grains ratio	−0.033	0.223	0.040	**0.660**	−0.197
Protein foods	0.053	0.012	**0.787**	−0.119	−0.078
Seafood, beans, and legumes	0.079	0.052	**0.772**	0.154	0.091
Dairy	**−0.733**	−0.020	0.051	0.037	0.061
Beverages	−0.083	**0.730**	−0.011	0.144	0.030
Fatty acid ratio	**0.648**	−0.071	0.190	0.098	0.188
Saturated fat	**0.775**	0.066	0.055	0.064	0.046
Added sugar	0.083	**0.763**	0.065	0.190	0.092
Sodium	−0.121	**−0.538**	−0.063	**0.569**	0.174

Loadings that are >0.5 are in bold.

**Figure 1:**
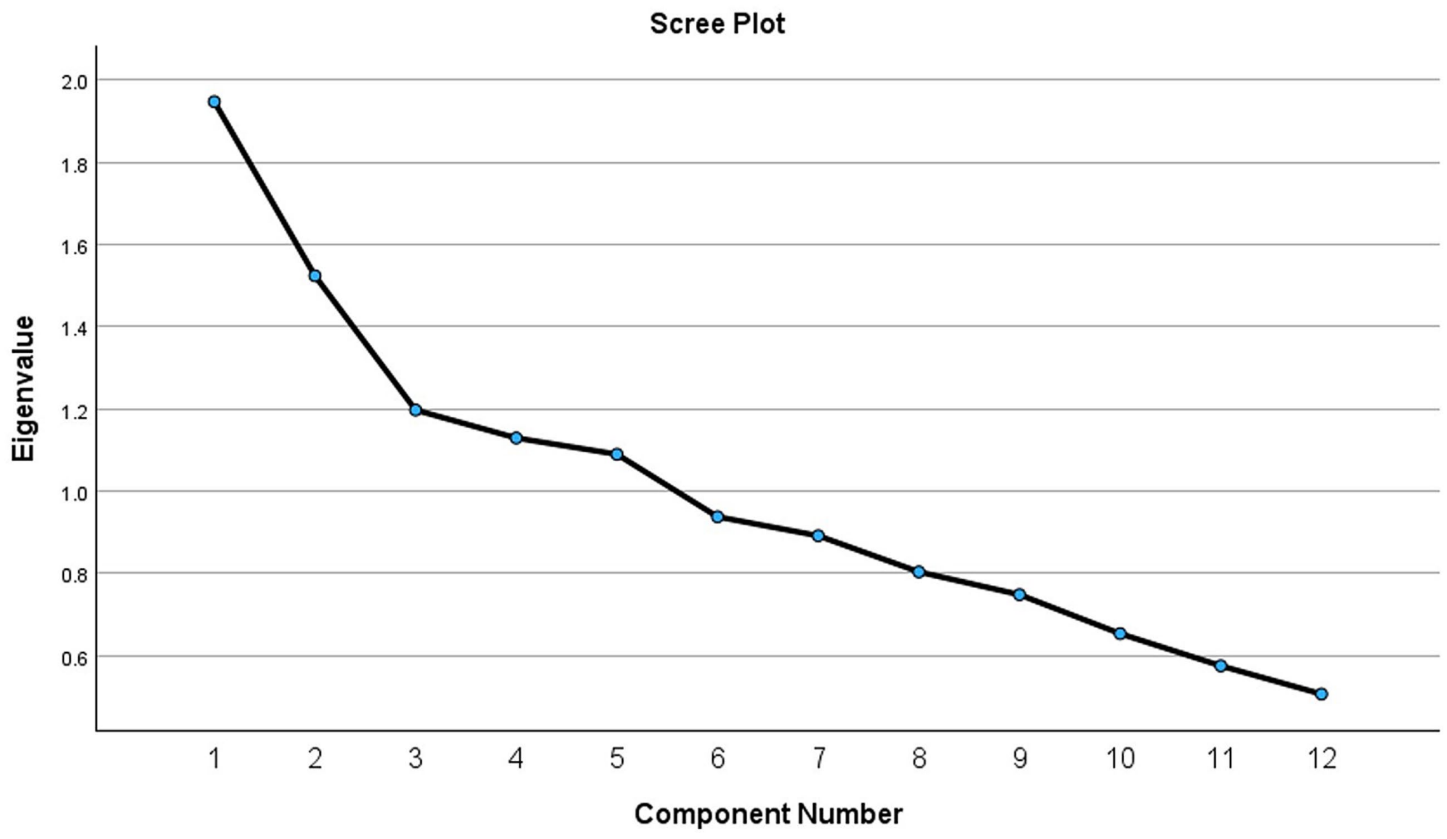
Principal component analysis scree plot of SHEI components.

The scree plot from the principal component analysis is presented in Figure 1. This multi-component structure supports the multidimensional nature of dietary intake and suggests that the SHEI captures at least five different aspects of diet quality.

#### 3.3.5 Scoring exemplary menus

The included exemplary menus are: the example menu in the SHPG-2024 and the 2000-calorie menu in the “Calorie Guide for Weight Loss” guide by the Saudi Ministry of Health, plus a DASH diet-based menu (27–29). As shown in Table 7, the yielded SHEI scores out of 100 were 80, 77, and 91, respectively.

**Table 7:** Exemplary menus from national sources scored using the SHEI.

Component	Menu source		
	SHPG-2024	Calorie guide for weight loss	DASH diet
Total SHEI score/100	80	77	91
Fruit/10	10.00	10.00	10.00
Vegetables/10	10.00	9.15	8.12
Grains/5	5.00	5.00	2.42
Whole grains ratio/5	5.00	0.78	5.00
Protein foods/5	3.50	5.00	4.86
Seafood, beans, and legumes/5	4.53	0.00	3.96
Dairy/10	10.00	10.00	7.29
Beverages/10	9.68	10.00	8.85
Fatty acid ratio/10	0.00	0.00	10.00
Saturated fat/10	4.15	7.10	10.00
Added sugar/10	10.00	10.00	10.00
Sodium/10	8.11	9.90	10.00

### 3.4 Concurrent validity

The SHEI and HEI-2015 scores showed a strong positive correlation (*r* = 0.867, *p* < 0.001), thereby supporting the concurrent validity of the SHEI as a diet quality assessment tool.

### 3.5 Reliability

#### 3.5.1 Internal consistency

The SHEI showed low internal consistency (Cronbach’s *α* = 0.237), which is expected given the diversity of the assessed components.

#### 3.5.2 Component correlations

The Spearman correlations among SHEI total, residual, and component scores were moderate to weak as reported in Table 8. The strongest correlations with the total SHEI score were observed for fruit (*r* = 0.542, *p* < 0.01) and saturated fat (*r* = 0.455, *p* < 0.01). For inter-component correlation, the strongest positive association was observed between added sugar and beverages (*r* = 0.466, *p* < 0.01), as well as fatty acid ratio and saturated fat (*r* = 0.362, *p* < 0.01). Notable negative correlations were found between saturated fat and dairy (*r* = −0.331, *p* < 0.01) and between dairy and fatty acid ratio (*r* = −0.245, *p* < 0.01). As for residual score correlations, added sugar demonstrated the strongest independent relationship (*r* = 0.222, *p* < 0.01). The weakest independent associations were observed for protein foods (*r* = 0.085, *p* < 0.05).

**Table 8:** Estimated Spearman correlation coefficients across total, residual, and component scores.

Component	SHEI Score	Fruit	Vegetables	Grains	Whole grains ratio	Protein foods	Seafood, beans, and legumes	Dairy	Beverages	Fatty acid ratio	Saturated fat	Added sugar	Sodium
SHEI Score	1.000												
Fruit	0.542**	1.000											
Vegetables	0.417**	0.046	1.000										
Grains	−0.005	−0.047	−0.161**	1.000									
Whole grains ratio	0.275**	0.129**	−0.018	0.092*	1.000								
Protein foods	0.170**	0.041	−0.012	−0.030	0.044	1.000							
Seafood, beans, and legumes	0.342**	0.032	0.054	−0.039	0.113**	0.253**	1.000						
Dairy	0.104**	−0.055	0.023	0.072	0.028	−0.112**	−0.040	1.000					
Beverages	0.280**	0.075*	0.080*	0.074*	0.141**	0.001	0.045	−0.052	1.000				
Fatty acid ratio	0.417**	0.035	0.126**	−0.145**	0.087*	0.148**	0.156**	−0.245**	−0.048	1.000			
Saturated fat	0.455**	0.170**	0.100**	−0.010	−0.017	0.049	0.135**	−0.331**	0.020	0.362**	1.000		
Added sugar	0.431**	0.220**	0.086*	−0.026	0.178**	0.038	0.086*	−0.101**	0.466**	0.097*	0.093*	1.000	
Sodium	0.138**	0.059	−0.037	−0.066	0.034	−0.039	0.041	0.037	−0.100**	−0.026	−0.096*	−0.178**	1.000
Residual score	-	0.202**	0.113**	−0.093*	0.165**	0.085*	0.170**	−0.207**	0.150**	0.150**	0.155**	0.222**	−0.088*

*p < 0.05, **p < 0.01.

## 4 Discussion

### 4.1 Summary of main findings

The psychometric evaluation of the SHEI demonstrated notable strengths in validity but limitations in reliability. Content validity was supported through alignment with SHPG-2024 recommendations during index development. Concurrent validity was demonstrated through a strong association with the well-established HEI-2015. Moreover, construct validity was supported in multiple ways: variability in total scores across individuals, variability in most component scores across quintiles, and between-group differences in terms of smoking status. Furthermore, it correlated poorly with energy intake and yielded moderate to high scores for exemplary menus. In terms of multidimensionality, it revealed five dimensions explaining 57.39% of the total variance. As for the evaluation of its reliability, its internal consistency was poor. Correlations across total, residual, and component scores showed highly variable results, ranging from moderate to low.

### 4.2 Population representativeness

This study included 702 participants from two cities, Riyadh in the central region and Al-Madinah in the western region, 62% women, with a median age of 27 (21–38) years. The sample was recruited through social media and skewed toward younger, urban, and more highly educated adults, which should be considered when generalizing the findings to the wider Saudi population. Comparable diet quality indices have typically been validated in larger study populations. For example, the HEI-2015 was evaluated using data from 7,935 NHANES participants (21), the HEFI-2019 using 20,103 adults from the Canadian Community Health Survey (25), and the CHEI using 12,473 participants from the China Health and Nutrition Survey (23). The only exception was the Netherlands’ DHD15-index, which used the longitudinal NQplus study, including 885 participants (24). This modest sample size is more comparable to the current analysis.

### 4.3 Construct validity

#### 4.3.1 Variability among individuals

Construct validity was supported through multiple analyses to ensure that the SHEI is a measure of diet quality as intended (33). This was examined by assessing how SHEI scores varied across individuals. The SHEI scores showed variability across the study sample, with total scores ranging from 37.4 in the lowest quintile to 68.2 in the highest quintile. Ceiling and floor effects occur when scores cluster at the maximum and minimum values, respectively, thereby limiting the sensitivity of the scale in detecting differences (34). Grains, protein foods, and beverage components maintained near-maximum scores across all quintiles, indicating a ceiling effect and strong adherence to SHPG-2024 recommendations. In contrast, sodium showed a floor effect, maintaining low scores across quintiles and suggesting poor adherence to sodium recommendations. These patterns align with established dietary habits in Saudi Arabia, where grains and proteins are commonly consumed and therefore achieve recommended amounts, in contrast to sodium intake which consistently exceeds recommended limits (35). Focusing on the mean total scores of DQIs across different countries, they ranged between 43.1 and 68.7 out of 100, with the SHEI (52.64) comparable to the CHEI (52.45) and HEI-2015 (56.6), suggesting that populations across diverse contexts generally achieve 50–60% of maximum scores (21, 23–25).

#### 4.3.2 Expected differences between groups

Another aspect is the variability in scores across specific population subgroups known to have differences in dietary patterns. In this analysis, smoking status was the only aspect that produced statistically significant results, with non-smokers having a SHEI score of (53.3 ± 0.5) compared to (48.8 ± 0.1) for smokers. Other socioeconomic factors, including sex, education level, and family income group, exhibited differences, but they were not significant. This may be due to the relatively narrow age range and generally high education level. International findings similarly show that DQI scores vary across socioeconomic and lifestyle factors, with consistently higher scores among non-smokers, women, older adults, those with higher education, and urban residents across the US, Canada, and China (21, 23, 25).

#### 4.3.3 Association with energy intake

The weak negative correlation between SHEI total scores and energy intake (*r* = −0.103, *p* = 0.006) is consistent with the SHEI measuring diet quality rather than diet quantity. Similarly, the HEI-2015 (*r* = −0.06), HEFI-2019 (*r* = −0.13), and CHEI (*r* = 0.10) all showed weak correlations with energy intake (21, 23, 25).

#### 4.3.4 Multidimensionality

Examining the multidimensional structure of DQIs is crucial to ensure they capture different aspects of diet quality (21–23, 25). Five dimensions were identified, explaining 57.39% of the total variance in SHEI component scores. However, due to the marginal KMO value (0.573), these dimensions should be interpreted with caution. Comparable multidimensional patterns have been reported for the HEI-2015 (4 dimensions), the HEFI-2019 (4 dimensions), and the CHEI (5 dimensions), supporting the multidimensional structure of DQIs (21, 23, 25).

#### 4.3.5 Scoring exemplary menus

A DQI should yield high scores when measuring exemplary menus based on recognized dietary guidelines (21–23, 25). The Saudi Ministry of Health’s Calorie Guide for Weight Loss menu received a score of 77, largely because it did not limit grain options to whole grains and included no items from seafood, beans, or legumes (27). The SHPG-2024 menu scored 80, with the reduction in its total score driven mainly by its higher saturated fat content, which affected both the saturated fat and fatty acid ratio components (29).

An international DASH diet-based menu scored 91, reflecting stronger alignment with SHEI recommendations (28). These differences demonstrate that the SHEI can distinguish between menus of varying alignment with dietary guidelines and highlight areas where Saudi guideline menus could be further strengthened, particularly regarding whole grains, seafood and legumes, and saturated fat recommendations. Across exemplary menus in other countries, scores were consistently high, ranging from 87.8–100 for the HEI-2015, 67.1 for the HEFI-2019, and 91.2–99.8 for the CHEI (21, 23, 25).

### 4.4 Concurrent validity

Since the HEI-2015 is the most established international DQI, it is often used to assess concurrent validity. The SHEI and HEI-2015 scores showed a strong positive correlation (*r* = 0.867), thereby supporting the concurrent validity of the SHEI. However, because both indices were scored using the same system and FPED components, this correlation may partly reflect shared methodology and overstate the expected agreement between independent measures. Likewise, the HEFI-2019 exhibited a positive correlation (*r* = 0.79) with the HEI-2015 (25).

### 4.5 Reliability

#### 4.5.1 Internal consistency

The Cronbach’s *α* values for the HEI-2015, HEFI-2019, and the CHEI were 0.6, 0.66, and 0.22, respectively (21, 23, 25, 36). Similar to the CHEI, the SHEI demonstrated very low internal consistency (Cronbach’s *α* = 0.237). Poor Cronbach’s α values are expected when evaluating DQIs and should not be interpreted negatively. This is because diet quality is multidimensional and adherence to dietary guidelines is highly variable among individuals (22). While the internal consistency of the SHEI is notably lower than that of the HEI-2015 and HEFI-2019, it remains comparable to the CHEI. These values could be interpreted as a characteristic of non-Western diets being more diverse.

#### 4.5.2 Component correlations

Weak to moderate correlation strengths were found between SHEI independent components and component-total correlations. The strongest significant correlations observed between components were for added sugar and beverages (*r* = 0.466), indicating possible overlap in measuring sugar intake in some beverages. Another correlation is between the fatty acid ratio and saturated fat components (*r* = 0.362), which may be because saturated fat is part of calculating the fatty acid ratio component. As for component–total correlations, the strongest residual association was observed for added sugar (*r* = 0.222), indicating that adherence to added sugar recommendations had the greatest independent influence on total scores. Similar correlation patterns between related components have been reported for the HEI-2015, HEFI-2019, and CHEI (21, 23, 25).

### 4.6 Strengths and limitations

In terms of strengths, this study presents the first efforts to evaluate the validity and reliability of a Saudi-specific diet quality assessment tool, using an evaluation framework aligned with internationally recognized DQIs. The dietary data were collected from two regions in Saudi Arabia, Riyadh in the central region and Al-Madinah in the western region, providing some geographical diversity. Data collection and entry were conducted by trained clinical dietitians. Whenever available, two-day 24-h dietary recalls were assessed for the majority of participants to better represent dietary habits.

The study has several limitations. First, the sample size was relatively small compared to similar international studies that typically use large nationally representative cohorts. Additionally, the sample was not equally distributed across regional or sociodemographic groups, which limits representativeness. National population-level dietary data could not be used, as they are not yet available in Saudi Arabia. Because the study applied several health-related exclusion criteria, the SHEI was not evaluated across the full range of clinical populations, where dietary needs differ, and diet quality is often most relevant. Future validation should therefore assess its performance in these groups and in more diverse samples.

A second critical limitation is the absence of a Saudi-specific FPED. No diet analysis software with Saudi food composition tables currently exists; therefore, relying on the U. S. FPED was unavoidable. Although it lacks some traditional Saudi foods, the diversity of the U. S. database meant that many Middle Eastern dishes commonly consumed in Saudi Arabia were included. To quantify this limitation, traditional foods with no close Western equivalents were flagged: 3.47% had no close match, and 6.34% had close nutritionally similar matches. Lastly, the presence of ceiling and floor effects suggests potential low sensitivity of the SHEI for some components.

## 5 Conclusion

This study presents a preliminary psychometric evaluation of a diet quality measurement tool specifically designed for Saudi adults, the SHEI. The evaluation of validity demonstrated construct and concurrent validity, with content validity established during index development. The evaluation of reliability exhibited weak to moderate component correlations and weak internal consistency reliability. Future validity and reliability studies of the SHEI should aim for better representation by addressing sample size and food database limitations. Further validation should also examine whether higher SHEI scores predict better health outcomes (e.g., lower obesity or reduced chronic disease incidence) in longitudinal studies. Despite its limitations, the SHEI provides a standardized tool that can support research and policy efforts in Saudi Arabia.

## Data Availability

The raw data supporting the conclusions of this article will be made available by the authors, without undue reservation.
